# Phenotype-Based Stratification of Olanzapine Response in Young Patients with Schizophrenia: Clinical, Neurochemical and Pharmacokinetic Insights

**DOI:** 10.1192/j.eurpsy.2026.11863

**Published:** 2026-07-31

**Authors:** W. Krzyściak, M. Pilecki, B. Bystrowska, N. Śmierciak, P. Karcz, E. Gomółka, A. Turek, M. Szwajca, A. Bryll, T. Popiela

**Affiliations:** 1 Department of Medical Diagnostics; 2 Department of Child and Adolescent Psychiatry and Psychotherapy, Chair of Psychiatry; 3 Department of Toxicology; 4 Department of Child and Adolescent Psychiatry; 5 Department of Electroradiology; 6 Toxicological Information and Laboratory Analysis Laboratory University Hospital, Jagiellonian University Medical College, Kraków; 7 Department of Child and Adolescent Psychiatry, Jagiellonian University Medical College, Krakow; 8 Chair of Radiology, Jagiellonian University Medical College, Kraków, Poland

## Abstract

**Introduction:**

Schizophrenia is a chronic psychiatric disorder involving dysregulation in dopamine, glutamate, and serotonin systems. Olanzapine is widely used in treatment, yet patient response varies significantly. Understanding how olanzapine and its metabolites relate to clinical symptoms and neurochemical markers may enable more personalized and effective therapeutic strategies.

**Objectives:**

To evaluate the clinical, neurochemical, and immunological impact of olanzapine and its metabolites in young schizophrenia patients, identify treatment response subgroups, and explore associations between neurotransmitter levels and symptom severity.

**Methods:**

Fifty-one patients (mean age: 27.7 years) diagnosed with schizophrenia and treated with olanzapine were included. Symptom severity was assessed using PANSS, CDSS, and BDI-II. Neurochemical and immunological parameters were evaluated.

Plasma concentrations of olanzapine and its metabolites were measured using a validated HPLC-MS/MS method (MassTox® TDM kit). The assay showed high linearity (1.034–145 μg/L, R² = 0.9998), precision (CV = 12.4%), and accuracy (bias: –2.7% to 4.2%).

Statistical analysis included partial correlation (adjusting for age, sex, treatment duration) and Hierarchical Clustering on Principal Components (HCPC). Clusters were compared using v-tests (*p* < 0.05).

**Results:**

Three phenotypic clusters were identified:

- **Cluster 1 (21.6%):** Patients responded well at low olanzapine levels (<4.35 μg/L), with significantly lower negative symptoms (PANSS: 11.91 vs. 21.22, *p* < 0.001) and depression scores (CDSS: 3.55 vs. 8.54, *p* = 0.001; BDI-II: 10.09 vs. 18.74, *p* = 0.008).
- **Cluster 2 (43.1%):** Despite high olanzapine levels (>4.35 μg/L), patients showed poor response, with elevated PANSS negative (24.73 vs. 21.22, *p* < 0.001) and total scores (91.45 vs. 79.88, *p* < 0.001), along with reduced glutamatergic activity in the PCC and ACC.
- **Cluster 3 (35.3%):** Younger patients (mean age: 21.2 years) with normal olanzapine levels but elevated glutamate and glutamine in the cingulate cortex, indicating potential early-stage hyperglutamatergic dysregulation.

Neurochemical correlations showed that glutamate positively correlated with psychotic symptoms (*r* = 0.29, *p* = 0.049), while glutamine correlated negatively (*r* = –0.29, *p* = 0.045). Serotonin correlated with negative symptoms (*r* = 0.32, *p* = 0.029).

**Conclusions:**

This study supports the feasibility of phenotype-driven treatment stratification in schizophrenia.

- **Cluster 1** patients may benefit from lower olanzapine doses.
- **Cluster 2** may require alternative strategies, such as glutamatergic modulation or switching to clozapine.
- **Cluster 3** could benefit from early neurochemical monitoring (e.g., MRS) to prevent progression.

Routine therapeutic drug monitoring (TDM), combined with neurochemical profiling, may improve treatment outcomes and minimize adverse effects in schizophrenia.

**Disclosure of Interest:**

None Declared

